# Genetic Diversity of Human Host Genes Involved in Immune Response and the Binding of Malaria Parasite in Patients Residing along the Thai-Myanmar border

**DOI:** 10.3390/tropicalmed6040174

**Published:** 2021-09-24

**Authors:** Kridsada Sirisabhabhorn, Wanna Chaijaroenkul, Kesara Na-Bangchang

**Affiliations:** 1 Graduate Program in Bioclinical Sciences, Chulabhorn International College of Medicine, Thammasat University (Rangsit Campus), Pathumthani 12121, Thailand; kridsiri@tu.ac.th (K.S.); cwanna@tu.ac.th (W.C.); 2 Center of Excellence in Pharmacology and Molecular Biology of Malaria and Cholangiocarcinoma, Thammasat University (Rangsit Campus), Pathumthani 12121, Thailand; 3 Drug Discovery and Development Center, Office of Advanced Science and Technology, Thammasat University (Rangsit Campus), Pathumthani 12121, Thailand

**Keywords:** *Plasmodium falciparum*, *Plasmodium vivax*, MCP1, TGFβ1, TNFα, IL4 (VNTR), IL6, IL10, TLR4, CD36, ICAM1

## Abstract

Polymorphisms of the genes encoding proteins involved in immune functions and the binding of malaria parasites to human host cells have been the focus of research in recent years, aiming to understand malaria pathogenesis and case severity and to exploit this knowledge to assert control over malaria. This study investigated the genetic diversity of the human host genes encoding proteins that are involved in immune functions and malaria parasite binding, i.e., MCP1 (−2518), TGFβ1 (−509), TNFα (−308), IL4 (VNTR), IL6 (−174), IL10 (−3575), TLR4 (299), CD36 (−188), and ICAM1 (469) in patients with mono-infection of *Plasmodium falciparum* and *Plasmodium vivax* infections in the multidrug-resistant areas along the Thai-Myanmar border. The association between gene polymorphisms and parasite density was also investigated. Genomic DNA (gDNA) of *P. falciparum* and *P. vivax* were extracted from whole blood and dried blood spot (DBS). Gene amplification and genotyping were performed by PCR and PCR-RFLP analysis, respectively. Of these samples, 178 and 209 samples were, respectively, mono-infection with *P. falciparum* and *P. vivax*. The ratio of *P. falciparum*: *P. vivax* was 46%:54%. Results showed marked variation in the frequency distribution and patterns of the genotypes and gene alleles of the nine immune response genes or human host genes. The SNPs of TGFβ1, IL10 and ICAM1, were significantly associated with *P. falciparum*, but not *P. vivax* parasite density. TGFβ1, IL10 and ICAM1, may play more significant roles in modulating *P. falciparum* than *P. vivax* parasitemia. The prevalence of the genotypes and gene alleles of these genes, including their association with parasite density, may vary depending on patient ethnicity and endemic areas. Information obtained from each endemic area is essential for treatment strategies and the development of vaccines for malaria prophylaxis in specific areas.

## 1. Introduction

Malaria remains one of the major global public health problems despite the declining incidence in recent years. In 2018, 3.2 billion people were at risk of malaria infection, with an estimated 219 million cases and 435,000 malaria-related deaths [1]. Over 90% of death cases were reported from sub-Saharan Africa, most of which were children under five years of age. Five species of malaria parasite infect humans, i.e., *Plasmodium falciparum*, *P. vivax*, *P. malariae*, *P. ovale*, and *P. knowlesi. P. falciparum* is the most virulent and widespread infectious species in tropical and subtropical countries due to the parasite’s resistance to the most available antimalarial drugs. The species *P. vivax* is the next most significant malaria species, which usually causes benign uncomplicated malaria with relapse from dormant hypnozoites in the liver. Its clinical features differ from those of *P. falciparum* malaria. Both *P. falciparum* and *P. vivax* often co-exist in several parts of the world. The signs and symptoms of malaria vary from uncomplicated to complicated or severe malaria. Severe malaria manifests life-threatening clinical signs and symptoms, which are defined according to criteria of the World Health Organization (WHO) as impaired consciousness, respiratory distress, multiple convulsions, circulatory collapse, abnormal bleeding, jaundice, hemoglobinuria, severe anemia, hypoglycemia, acidemia, hyperlactatemia, renal impairment, and hyperparasitemia (>250,000 parasites/µL) [2]. Cerebral malaria is one of the most significant forms of severe malaria involving the two major pathogenesis processes, i.e., cytoadherence and immunogenic reactions. Most cases of cerebral malaria are due to *P. falciparum* infection, whereas *P. vivax* infection rarely causes cerebral malaria [3].

Both parasite and human host-related factors play significant roles in malaria susceptibility, pathogenesis, and disease severity [4]. Among the host-related factors, polymorphisms in the genes encoding proteins and involved in either immune functions (MCP1, TGFβ1, TNFα, IL4 VNTR, IL6, and IL10), or parasite binding to human host cells (TLR4, ICAM1, and CD36) have been the focus of research in recent years as researchers try to understand malaria pathogenesis and severity and to exploit the knowledge for malaria control. Results of various reports for the relationship between the host genetic polymorphisms and malaria susceptibility, pathogenesis and disease severity, however, are conflicting, depending on endemic areas and observation periods under investigation. The present study investigated the genetic diversity of the human host genes involved in immune response [monocyte chemoattractant protein 1 (MCP1)-2518, transforming growth factor β1 (TGFβ1)-509, tumor necrosis factor α (TNFα)-308, interleukin 4 (IL4) variable number tandem repeat (VNTR), interleukin 6 (IL6)-174, and interleukin 10 (IL10)-3575], as well as those involved in malaria parasite binding [toll-like receptor 4 (TLR4)-299, cluster of differentiation 36 (CD36)-188, and intercellular adhesion molecule 1 (ICAM1)-469] in patients with mono-infection of *P. falciparum* or *P. vivax*, residing in the multidrug-resistant areas along the Thai-Myanmar border [5]. The polymorphisms of the MCP1, TGFβ1, TNFα, IL4 VNTR, IL6, IL10 and CD36 genes involve nucleotide positions located in the promotor region, and the numbers indicate nucleotide positions. The polymorphisms of the TLR4 and ICAM1 genes involve amino acid changes, and the numbers indicate amino acid positions. The association between the polymorphisms (single nucleotide polymorphisms: SNPs) of these genes at specific amino acid/nucleotide positions and parasite density, as a criterion of malaria severity, was also investigated.

## 2. Materials and Methods

### 2.1. Samples and Study Site

The study was conducted in 2007 and 2015 at malaria clinics within the two malaria-endemic areas located along the Thai-Myanmar border, i.e., Mae-Sot (Tak province) and Sai Yok (Kanchanaburi province) districts. Approval for the study protocol was obtained from the Ethics Committee for Research in Human Subjects, Ministry of Public Health of Thailand (Number 3/52-293) and the Biosafety Committee of Thammasat University, Thailand (Number 082/2560). The minimum sample size of 385 patients was estimated based on the prevalence of ICAM-1 polymorphism, which was the largest sample size among other gene polymorphisms [6].

Whole blood and dried blood spot (DBS) samples were collected from patients with confirmed infections with *P. falciparum* or *P. vivax* by microscopic examination of Giemsa’s thin blood smears and nested PCR [7]. The parasite density of each sample was determined according to WHO guidelines [8]. Parasite density (per µL blood) in all samples was determined from thick blood smears under a light microscope at 1000× magnification. The number of asexual stage parasites, i.e., rings, trophozoites, and schizonts were counted (three times) against 200 white blood cells (WBC) in the thick blood film. Parasite density (per microliter) was calculated as the number of parasites multiplied by the number of WBCs and divided by 200. Whole blood samples were stored at −20 °C and transferred together with DBS samples to the Center of Excellence in Pharmacology and Molecular Biology of Malaria and Cholangiocarcinoma, Thammasat University, Thailand.

### 2.2. Preparation of Genomic DNA

Genomic DNA (gDNA) of whole blood and DBS samples infected with *P. falciparum* or *P. vivax* (mono-infection) were extracted using QIAamp DNA Blood Mini kit (QIAGEN, Hilden, Germany).

### 2.3. Gene Amplification by Polymerase Chain Reaction (PCR)

The PCR conditions (total volume of 25 µL) were set as follows: 5 µL of sample gDNA, 1.5 µL of MgCl_2_, 1.5 µL of 10 × KCl buffer, 0.5 µL of dNTP, 0.25 µL of *Taq* polymerase enzyme, and 0.5 µL each of forward and reverse primers. The primers used in the reaction are presented in Table 1. Gene amplification, denaturation, and extension steps (35 cycles) were set as 95 °C for 1 min and 72 °C for 1 min. For the annealing step, the temperature gradient specific to each gene was set at 40–60 °C. The amplified gene product was separated by 1.5% agarose gel electrophoresis.

### 2.4. Identification of SNPs and Genotypes by Restriction Fragment Length Polymorphism (RFLP)

Detailed information of the polymorphic points, amino acid changes, genotypes, and analysis conditions of all nine genes investigated are summarized in Table 1. The genetic polymorphism of TLR4 results from an amino acid change from aspartate to glycine at the nucleotide position 896, while that of ICAM-1 results from an amino acid change from lysine to glutamine at the nucleotide position 469. The reaction mixture (20 µL) consisted of 10 µL of PCR amplicon, 2.0 µL of the specific buffer, 7.9 µL of distilled water, and 1 U of specific restriction enzyme of individual SNP. The mixture was incubated at 37 °C and the RFLP products were analyzed by electrophoresis on 3% agarose gel.

### 2.5. Statistical Analysis

The frequency of genotypes and alleles of each gene are presented as number and percentage (%) values. Quantitative data are summarized as median (range) values. The deviation of gene distribution was determined by Hardy–Weinberg Equilibrium (HWE) (https://dr-petrek.edu>documents>HWE.xls, accessed on 10 January 2021). The distribution patterns of gene types, genotypes, and gene alleles were analyzed using the Chi-square test. The association between genotypes and gene alleles and *P. falciparum* and *P. vivax* parasite density was determined using Kruskal–Wallis and Mann–Whitney U tests. The statistical significance level was set at α = 0.05.

## 3. Results

**Figure 1:**
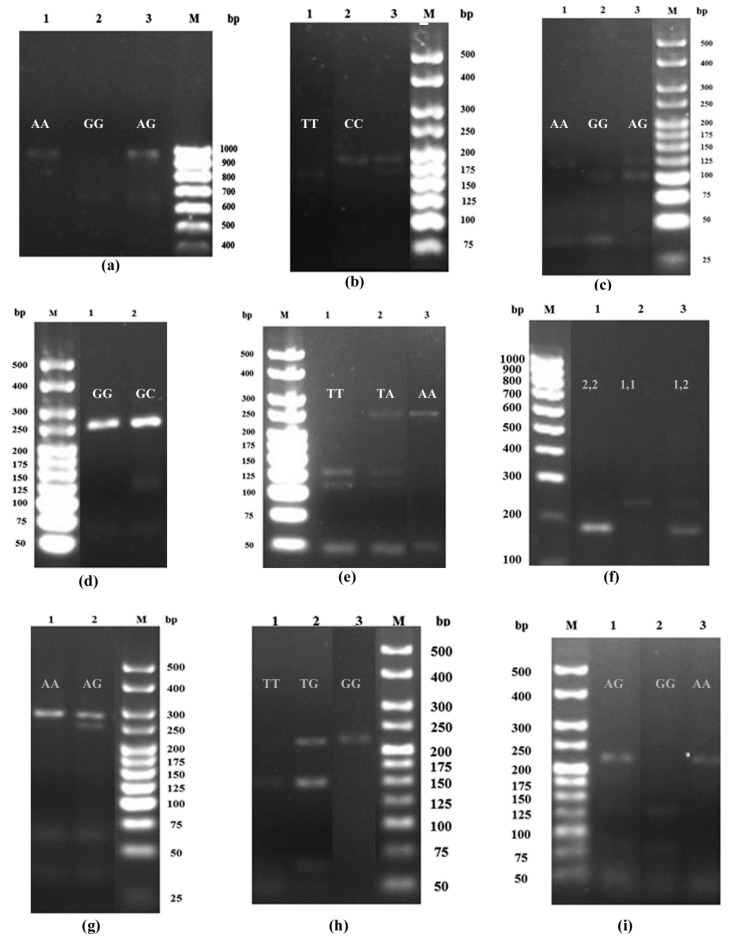
The PCR-RFLP products for (**a**) MCP1 polymorphism at position −2518: AA (940 bp), GG (650 bp) and GA (940 and 650 bp) genotypes, (**b**) TGFβ1 polymorphism at position −509: CC (178 bp), TT (159 bp) and TC (178 and 159 bp) genotypes, (**c**) TNFα polymorphism at position −308: AA (117 bp), GA (117 and 97) and GG (97 bp) genotypes, (**d**) IL6 polymorphism at position −174: GG (233 bp) and GC (233 and 122 bp) genotypes, (**e**) IL10 polymorphism at position −3575: AA (228 bp), TA (228, 121 and 107 bp) and TT (121 and 107 bp) genotypes, (**f**) IL4 VNTR: 1,1 (253 bp), 2,2 (183 bp) and 1,2 (253 and 183 bp) genotypes, (**g**) TLR4 polymorphism at position 299: AA (249) and AG (249 and 223 bp) genotypes, (**h**) CD36 polymorphism at position −188: GG (213 bp), TG (213, 148 bp) and TT (148) genotypes, and (**i**) ICAM1 polymorphism at position 469: AA (223 bp), GG (136), and AG (223, 136) genotypes.

A total of 387 samples (71 whole blood and 316 DBS samples) were included in the analysis. This number comprised 172 and 215 samples obtained from Tak, and Kanchanaburi province, respectively. Most 96.6% of patients were Burmese, and 3.4% were ethnic minorities. Totals of 178 (46.0%) and 209 (54.0%) samples were mono-infection with either *P. falciparum* or *P. vivax*, respectively. Successful amplification of the nine genes under investigation varied between 24.2–100.0% and 61.2–100.0% for *P. falciparum* and *P. vivax*-infected samples, respectively. Representative images of the PCR-RFLP products of the nine polymorphic genes under investigation are shown in Figure 1. The distribution of *P. falciparum*- and *P. vivax*-infected cases, including the parasite density in the two endemic areas, are summarized in Table 2.

### 3.1. Genotype and Allele Distribution

The distribution patterns of genotypes and gene alleles of the nine genes in patients infected with *P. falciparum* and *P. vivax* are summarized in Table 3, respectively. The predominant genotypes of most genes in patients with *P. falciparum* infection were homozygous wild type/mutant genotypes, with the exception of MCP1 and CD36, of which the predominant genotypes were heterozygous. The dominant alleles of most genes were wild type alleles (with a frequency of up to 100.0%), with the exception of IL4VNTR, of which the predominant allele was two gene repeats. The genotypes and gene alleles of most genes showed a significant difference in distribution patterns. For *P. vivax*, similarly to *P. falciparum* isolates, the predominant genotypes for most genes were homozygous wild type, with the exception of MCP1 and TNFα1, of which the predominant genotypes were heterozygous. The dominant alleles of most genes were wild type alleles (with a frequency of up to 99.0%), with the exception of IL4VNTR, of which the predominant allele was two gene copies. The genotypes but not gene alleles of patients infected with *P. vivax* showed a significant difference in distribution patterns. The distribution patterns of genotypes of the samples obtained from the malaria-infected patients significantly deviated from Hardy–Weinberg Equilibrium (HWE), i.e., IL10 (*p* = 0.006), ICAM1 (*p* = 0.015), TGFβ1 (*p* = 0.027), and CD36 (*p* < 0.001) in *P. falciparum*-infected patients and TNFα1 (*p* < 0.001), IL10 (*p* < 0.001), and ICAM1 (*p* = 0.003) in *P. vivax*-infected patients.

### 3.2. Association between Genotypes/Gene Alleles and Parasite Density

**Figure 2:**
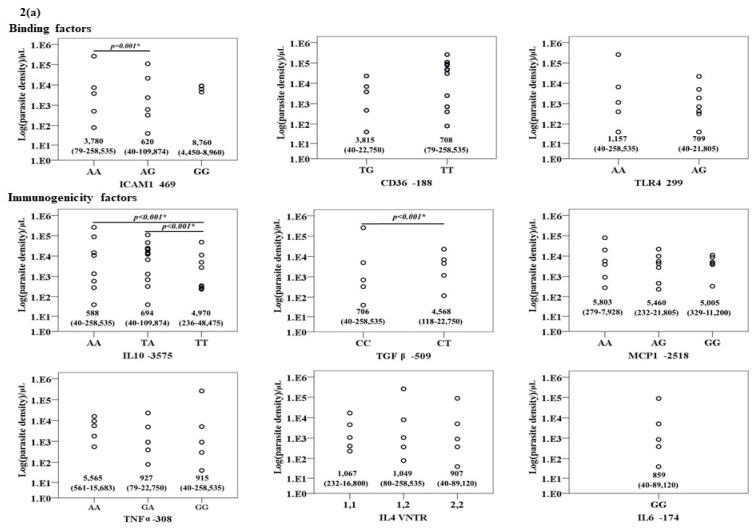

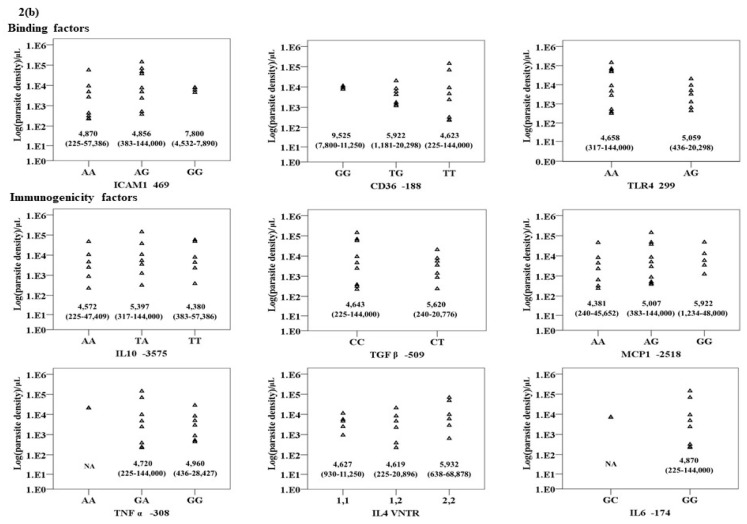
The relationship between the genotypes of the nine genes under investigation and Log (parasite density) (/μL) in (**a**) *P. falciparum* and (**b**) *P. vivax* infections.

The association between the genotypes of the nine genes in patients with *P. falciparum* and *P. vivax* infections are summarized in Figure 2a,b. Significant associations were observed for TGFβ1, IL10, and ICAM1 genotypes in patients with *P. falciparum* infection. The parasite density in patients with the heterozygous genotype for TGFβ1 was significantly higher than those with the wild type genotypes [median (range): 4568 (118–22,750) vs. 706 (40–258,535) /μL)]. Conversely, the parasite density in the isolates with the heterozygous or homozygous mutant genotype for IL10 was significantly lower than the wild type genotype [median (range): 588 (40–258,535) vs. 694 (40–109,874) vs. 4,970 (236–48,475) /μL)]. The parasite density in patients carrying the heterozygous genotype of ICAM1 was significantly lower than the wild type genotype [median (range): 620 (40–109,874) vs. 3780 (79–258,535) /μL)]. No association between genotypes and *P. vivax* parasite density was found.

For the gene alleles, similarly to those observed with genotypes, significant associations were observed between the gene alleles of TGFβ1, IL10 and ICAM1, and parasite density in patients with *P. falciparum* infection. The parasite density in the isolates carrying the mutant allele of TGFβ1 was significantly higher than those with the wild type allele [median (range): 4568 (118–22,750) vs. 1109 (40–258,535) /µL)]. Conversely, the parasite density in patients carrying heterozygous alleles of IL10 and ICAM1 was significantly lower than those with the wild type allele [median (range): 623 (40–258,535) vs. 2300 (40–109,874) /µL and 627 (40–109,874) vs. 1109 (40–258,535) vs. /µL, respectively]. No association between genotypes and *P. vivax* parasite density was found.

## 4. Discussion

The frequencies and distribution patterns of genotypes and gene alleles of most genes under investigation were markedly different, depending on the species of the malaria parasite. It is noteworthy that results may suggest a more significant contribution of these genes in patients with *P. falciparum* compared with *P. vivax* infection for malaria parasite density, one of the criteria for malaria severity. The HWE deviation observed with TGFβ1, TNFα, IL10, CD36 and ICAM1 could be due to gene mutation, population migration, and/or selection of genotypes/alleles of the study populations. In the present study, the SNPs of the two host genes involved in immune functions, i.e., TGFβ1 and IL10, and the one involved in malaria parasite binding (ICAM1) were significantly associated with *P. falciparum* parasitemia. The SNPs of TGFβ1 (CT genotype) was significantly associated with high parasite density, whereas that of IL10 (A allele and TA and AA genotypes) and ICAM1 (G allele and AG genotype) were significantly associated with low parasite density compared with their corresponding wild type alleles/genotypes. However, this association was not observed in patients with *P. vivax* infection. This may suggest the significant contribution of these genes in host susceptibility to infection and disease severity in *P. falciparum* cases but not *P. vivax*.

The CC genotype of TGFβ has been reported to link with low parasite density in *P. falciparum*, while the CT genotype is linked with high parasite density. It is possible that the homozygous genotype (CC) results in the decrease in TGFβ cytokine production for malaria eradication [20], whereas the heterozygous genotype (CT) results in the increase in TGFβ cytokine production and high parasite density, thereby, perhaps, enhancing the risk of severe malaria [21]. The T allele may play a more significant contribution to treatment outcomes than the C allele, and anchoring T allele may suppress the function of C allele for TGFβ level production [22,23,24] through enhancing promoter transcription at the Yin-Yang1 binding site [25]. Nevertheless, some studies reported the association between low TGFβ1 level and severe clinical outcomes in both *P. falciparum* and *P. vivax* infections [26,27,28].

For IL10 gene, the A allele and TA and AA genotypes were associated with low parasite density in patients with *P. falciparum* infection compared with the wild type genotype/allele. It is hypothesized that the T allele and TT genotype may increase the production of IL10 and severe malaria [29,30]. This, in turn, suppresses the production of IL10 by IL10 producing cells [31,32], which alleviates the action of this cytokine on reducing malaria parasitemia, and thus the risk of hyperparasitemia. An opposite effect may occur with the A allele and AA and TA genotypes. Our results support the results from a previous study showing a more significant association between IL10 -3575A and low plasma IL10 level compared with the -3575 T allele [33]. In addition, a significant association was found between IL10 -1082A and decreased IL10 production in severe malaria [34]. It is likely that these allele/genotypes result in the reduction of IL10 production [16,32], thereby triggering the production of this cytokine by the target cells [35]. For *P. vivax* infection, in spite of the high plasma levels of IL10 and TGFβ during the infection phase [36], minimum parasite density over the threshold level is required for pathogenesis induction [37]. *Plasmodium vivax* has a hypnozoite stage, mono-invasion and long incubation period of the asexual stage. These factors result in low parasitemia and pathogenicity [38]. Since *P. vivax* infection produces non-severe symptoms, patients usually do not seek treatment with antimalarial drugs. This may lead to chronic morbidity and high plasma cytokine levels. The association between human genetics and plasma levels of these cytokines remains conflicting. The result of the current study in the Thai-Myanmar area, however, showed no association between the genetic polymorphisms of the human immune cytokines and *P. vivax* parasite density.

The adhesion of infected red blood cells to the microvasculature of the brain is a key feature of cerebral malaria [39,40]. The host binding molecule ICAM1 or CD54 is located on the surface of endothelial cells. It acts as a binding receptor of the parasite virulence protein *Pf*EMP1 (*Plasmodium falciparum* erythrocyte membrane protein 1), leading to an enhanced risk of severe clinical outcomes [40]. In the present study, the G allele and AG genotype were associated with low *P. falciparum* density. The mutation of the ICAM1 gene at this position may result in an impaired binding property of the ICAM1 molecule on the infected erythrocytes, which was supported by the results of an in vivo study [41]. This leads to a low risk of parasite cytoadherence, thereby, decreasing the severity of malaria pathogenesis [42,43]. However, some studies reported conflicting results [44]. For *P. vivax*, the parasite exports *vir* protein encoded by *vir* gene (variant interspersed repeats gene), which binds to ICAM1 receptor molecule. Unlike *Pf*EMP1, *vir* protein is not a potent cytoadherence and sequestering ligand [45,46]; therefore, severe disease manifestation is unlikely. Our result supports no association between the polymorphisms of these parasite binding genes and susceptibility to high *P. vivax* parasitemia in the Thai-Myanmar border area.

There was no significant association between *P. falciparum* or *P. vivax* parasite density and SNPs of the MCP1, TNFα, IL4, IL6, IL10, TLR4, or CD36 genes. Current reports on the association between malaria parasite density and severity and the SNPs of these genes are controversial. High MCP1 levels during *P. falciparum* and *P. vivax* infection were associated with severe malaria and mortality [47,48,49,50,51,52]. The IL4 VNTR 1R allele was associated with severe malaria with increased plasma IL4 level [53] and reduced risk of severe malaria, while the IL4 VNTR 2R allele was associated with decreased plasma IL4 level [16]. The SNP of IL6 (-174GC) was associated with hyperparasitemia, malaria severity and IL6 level [14,54]. The TLR4 SNP (299AG) was associated with severe anemia and hyperparasitemia in Ghanaian children [55,56]. A heterozygous genotype (TG) of CD36 was protective against severe malaria in India [57,58].

It was noted that hyperparasitemia is only one criterion for malaria severity. Malaria symptoms and disease severity can be influenced by many factors such as parasite mixed strain infection, endemic malaria area, host genetic background, time of infection (the duration from infection and sample collection), medication history, nutrition, prior infections, and co-infection with other pathogens (viruses and bacteria). These factors could be confounding factors for conflicting results reported in this as well as other studies in other malaria-endemic areas. This should be considered when analyzing and interpreting the results. An additional limitation of the current study is that the number of samples used in each association analysis is small, particularly for the TNFα and MCP1 genes. Information obtained from each endemic area, therefore, is essential for treatment strategies and the development of vaccines for malaria prophylaxis in those specific areas. Furthermore, other potential genes were identified in the large-scale genome-wide association studies, e.g., gene expression profiles related to neutrophil and erythroid activity, sickle cell hemoglobin S variant, interleukin receptors and kelch-like protein KLHL3 [59,60,61], should be included in the analysis.

## 5. Conclusions

The genes involved in host immune response and binding of the malaria parasite, particularly TGFβ1, IL10 and ICAM1 may play a more significant role in malaria parasitemia in *P. falciparum* than in *P. vivax* infection. The SNPs of these genes were associated with *P. falciparum* parasite density. The prevalence of the genotypes and alleles of these genes reported in various studies, including their association with malaria parasite density and disease severity, is conflicting due to confounding factors. Interpretation and conclusion on the association of the polymorphisms of genes involved in host immune response and binding of the malaria parasite and *P. falciparum* and *P. vivax* parasitemia should be based on studies conducted in each endemic area with adequate sample size and minimized confounding factors.

## Figures and Tables

**Table 1:** The primers and analysis conditions of nine host genes involved in immune response and malaria parasite binding (amino acid/nucleotide positions with mutation and genetic analysis) under investigations.

No.	Gene	Amino Acid Position	Mutation	NCBI Number	Primer Sequence (5′–3′)	Restriction Enzyme	Genotype	Description	Fragment bp.	Ref.
1	MCP-1	−2518	A > G	rs1024611	Forward: 5′-CTTTCCCTTGTGTGTCCCC-3′ Reverse: 5′-TTACTCCTTTTCTCCCCAACC-3′	*PvuII*	AA	Homozygous wild type	940	[6]
							AG	Heterozygous mutant	940, 650, 290	
							GG	Homozygous mutant	650, 290	
2	TGFβ-1	−509	C > T	rs1800469	Forward: 5′-GTCCCTCTGGGCCCAGTTTC-3′ Reverse: 5′-GAGGGGGCAACAGGACACCTTA-3′	*Afl II*	CC	Homozygous wild type	178	[9,10]
							CT	Heterozygous mutant	178, 159, 19	
							TT	Homozygous mutant	159, 19	
3	TNFα	−308	G > A	rs1800629	Forward: 5′-GGCAATAGGTTTTGAGGGCCATG-3′ Reverse: 5′-CACACTCCCCATCCTCCCTGATC-3′	*NcoI*	GG	Homozygous wild type	97, 20	[11]
							GA	Heterozygous mutant	117, 97, 20	
							AA	Homozygous mutant	117	
4	IL4 VNTR	variable number of tandem repeat (VNTR)	1 repeat	rs8179190	Forward: 5′-TAGGCTGAAAGGGGGAAAGC-3′ Reverse: 5′-CTGTTCACCTCAACTGCTTCC-3′	none	1,1	Homozygous variant	253	[12,13]
			2 repeats				2,2	Homozygous variant	183	
			1, 2 repeats				1,2	Heterozygous variant	253, 183	
5	IL-6	−174	G > C	rs1800795	Forward: 5′-TTGTCAAGACATGCCAAAGTGCT-3′ Reverse: 5′-GCCTCAGACATCTCCAGTCC-3′	*NlaIII*	G allele	Wild type allele	233, 57, 13	[14,15]
							C allele	Mutant allele	122, 111, 57, 13	
6	IL-10	−3575	T > A	rs1800890	Forward: 5′-GGTTTTCCTTCATTTGCAGC-3′ Reverse: 5′-ACACTGTGAGCTTCTTGAGG-3′	*ApoI*	TT	Homozygous wild type	121, 107	[16]
							TA	Heterozygous mutant	228, 121, 107	
							AA	Homozygous mutant	228	
7	TLR-4	299 *	A > G	rs4986790	Forward: 5′-GATTAGCATACTTAGACTACTACCTCCATG-3′ Reverse: 5′-GATCAACTTCTGAAAAAGCATTCCCAC-3′	*NcoI*	AA	Homozygous wild type	249	[17]
							AG	Heterozygous mutant	223, 26	
							GG	Homozygous mutant	26	
8	CD-36	−188	T > G	rs3211938	Forward: 5′-CTATGCTGTATTTGAATCCGACG-3′ Reverse: 5′-ATGGACTGTGCTACTGAGGTTAT-3′	*NdeI*	TT	Homozygous wild type	148, 65	[18,19]
							TG	Heterozygous mutant	213, 148, 65	
							GG	Homozygous mutant	213	
9	ICAM-1	469 **	A > G	rs5498	Forward: 5′-GGAACCCATTGCCCGAGC-3′ Reverse: 5′-GGTGAGGATTGCATTAGGTC-3′	*BstUI*	KK (AA)	Homozygous wild type	223	[6]
							KE (AG)	Heterozygous mutant	223, 136, 87	
							EE (GG)	Homozygous mutant	136, 87	

* Amino acid change from aspartate to glycine at the nucleotide position 896. ** Amino acid change from lysine to glutamate at the nucleotide position 469.

**Table 2:** Demographic data of samples collected for genetic analysis. Data are presented as number (%) or median (range) value.

Site	Total	*P. falciparum*	*P. falciparum* Parasitemia	*P. vivax*	*P. vivax* Parasitemia
	N	n (%)	Median (Range)	n (%)	Median (Range)
Tak	172	147 (82.6)	828 (40–258,535)	25 (12.0)	6890 (2340–13,200)
Kanchanaburi	215	31 (17.4)	9346 (232–109,874)	184 (88.0)	4532 (225–144,000)
Total	387	178 (46.0)	1135.5 (40–258,535)	209 (54.0)	4870 (225–144,000)

**Table 3:** Distribution of genotypes and gene alleles of host genes related to malaria severity. Data are presented as number (%) or median (range) value.

***P. falciparum* Infection.**							
**Gene Type**	**Gene**	**Polymorphism**	**Genotype**			**Gene Allele** **n (%)**	
			**Homozygous Wild Type** **n (%)**	**Heterozygous Genotype** **n (%)**	**Homozygous Mutant** **n (%)**		
Immune functions	MCP1	−2518	AA: 14 (32.6)	AG:19 (44.2)	GG: 10 (23.3)	A: 47 (54.7)	G: 39 (45.3)
	TGFβ1	−509	CC: 125 (71.4)	CT: 50 (28.6)	TT: 0 (0.0)	C: 300 (85.7)	T:50 (14.3)
	TNFα1	−308	GG: 92 (60.1)	GA: 58 (37.9)	AA: 3 (2.0)	G: 242 (79.1)	A: 64 (20.9)
	IL6	−174	GG: 119 (100.0)	GC: 0 (0.0)	CC: 0 (0.0)	G: 238 (100.0)	C: 0 (0.0)
	IL10	−3575	TT: 65 (38.9)	TA:64 (38.3)	AA: 38 (22.8)	T: 194 (58.1)	A: 140 (41.9)
	IL4	VNTR	1,1 repeat: 8 (5.5)	1,2 repeats: 44 (30.1)	2,2 repeats: 94 (64.4)	-	-
Malaria parasite binding	TLR4	299	AA: 167 (96.0)	AG: 7 (4.0)	GG: 0 (0.0)	A: 341 (98.0)	G: 7 (2.0)
	CD36	−188	TT: 78 (46.2)	TG: 91 (53.8)	GG: 0 (0.0)	T: 247 (73.1)	G: 91 (26.9)
	ICAM1	469	AA: 103 (57.9)	AG: 72 (40.4)	GG: 3 (1.7)	A: 278 (78.1)	G: 78 (21.9)
***P. vivax* Infection.**							
**Gene Type**	**Gene**	**Polymorphism**	**Genotype**			**Gene Allele** **n (%)**	
			**Homozygous Wild Type** **n (%)**	**Heterozygous Genotype** **n (%)**	**Homozygous Mutant** **n (%)**		
Immune functions	MCP1	−2518	AA: 63 (40.4)	AG: 68 (43.6)	GG: 25 (16.0)	A: 194 (62.2)	G: 118 (37.8)
	TGFβ1	−509	CC: 170 (82.9)	CT: 35 (17.1)	TT: 0 (0.0)	C: 375 (91.5)	T: 35 (8.5)
	TNFα1	−308	GG: 65 (36.7)	GA: 111 (62.7)	AA: 1 (0.6)	G: 241 (68.1)	A: 113 (31.9)
	IL6	−174	GG: 181 (99.5)	GC: 1 (0.5)	CC: 0 (0.0)	G: 363 (99.7)	C: 1 (0.3)
	IL10	−3575	TT: 76 (49.7)	TA: 27 (17.6)	AA: 50 (32.7)	T: 179 (58.5)	A: 127 (41.5)
	IL4	VNTR	1,1 repeat: 9 (7.0)	1,2 repeats: 42 (32.8)	2,2 repeats: 77 (60.2)	-	-
Malaria parasite binding	TLR4	299	AA: 157 (91.8)	AG: 14 (8.2)	GG: 0 (0.0)	A: 328 (95.9)	G: 14 (4.1)
	CD36	−188	TT: 140 (80.0)	TG: 33 (18.9)	GG: 2 (1.1)	T: 313 (89.4)	G: 37 (10.6)
	ICAM1	469	AA: 119 (56.9)	AG: 87 (41.6)	GG: 3 (1.4)	A: 325 (77.8)	G: 93 (22.2)

## Data Availability

Data will be made available upon request.
